# *Epimedium* attenuates neuroinflammation and ameliorates Alzheimer's disease through a KAT2B-dependent mechanism

**DOI:** 10.3389/fmed.2026.1811410

**Published:** 2026-04-20

**Authors:** Jie Gao, Ming Lang Song, Jie Liu, ZhiBin Jiang, Wen Li, YunZhi Chen

**Affiliations:** Basic Chinese Medicine School (Qihuang College), Guizhou University of Traditional Chinese Medicine, Guiyang, Guizhou, China

**Keywords:** Alzheimer's disease, *Epimedium*, KAT2B, neuroinflammation, NF-κB, Traditional Chinese Medicine Systems Pharmacology

## Abstract

**Background:**

Alzheimer's disease (AD) is the leading cause of dementia worldwide, yet effective therapies for this neurodegenerative disorder remain scarce. *Epimedium*, a herb with a history of thousands of years in traditional Chinese medicine, exhibits diverse biological activities and holds potential therapeutic effects against Alzheimer's disease. However, studies on its modern pharmacological mechanisms remain relatively limited.

**Methods:**

TCMSP and PubChem were used to retrieve *Epimedium* constituents, and SwissTargetPrediction was used to predict potential targets. GeneCards, OMIM, and GEO databases were used to identify targets associated with AD. The AD model was established by intragastric administration of AlCl3 combined with intraperitoneal injection of D-galactose, with the normal saline group serving as controls. Cognitive function was assessed by the Morris water maze, and histopathological changes were observed by H&E staining. ELISA detected IL-1β and TNF-α levels, and qRT-PCR detected KAT2B, NF-κB, IL-1β, and TNF-α expression.

**Results:**

We identified 172 key targets, with KAT2B and ACACB determined as core genes through transcriptomic screening. *In vivo* studies showed that *Epimedium* significantly ameliorated cognitive deficits and alleviated histopathological damage in hippocampal neurons. Furthermore, *Epimedium* suppressed neuroinflammation by reducing IL-1β and TNF-α levels and modulated the mRNA expression of key targets (KAT2B, NF-κB) in the hippocampus.

**Conclusion:**

*Epimedium* improves pathological alterations in the hippocampal tissue and alleviates cognitive and behavioral impairments in rats by modulating neuroinflammation through KAT2B, thereby providing a scientific basis for its potential application and development as a therapeutic agent.

## 1 Introduction

Alzheimer's disease (AD) is a devastating, insidious, and irreversible neurodegenerative disorder characterized by the chronic and progressive deterioration of the central nervous system (CNS) (1). According to recent epidemiological reports from the World Health Organization (WHO), approximately 25% to 30% of individuals aged 85 and older exhibit clinical manifestations of cognitive decline (2). Global projections indicate a looming public health crisis, with the prevalence of dementia expected to double in Europe and triple worldwide by 2050 (3).

The canonical neuropathological landscape of AD is defined by the extracellular deposition of amyloid-β (Aβ) into senile plaques and the intracellular accumulation of hyperphosphorylated tau protein as neurofibrillary tangles (NFTs) (4). These proteopathic aggregates trigger a multifaceted pathological cascade, encompassing oxidative stress, neuroinflammation, and programmed cell deat (5), which collectively culminate in the disruption of neural circuitry and the inexorable decline of cognitive function (6, 7).

Crucially, an emerging body of evidence has repositioned neuroinflammation as a fundamental driver of AD pathogenesis, recognized now as the third core pathological hallmark alongside Aβ plaques and tauopathy (8). This inflammatory milieu is primarily orchestrated by the aberrant activation of microglia and astrocytes, which release a pleiotropic array of pro-inflammatory mediators (9, 10). In the context of AD, neuroinflammation exhibits a dichotomous, dual-phase profile: while transient microglial activation initially serves a neuroprotective role by facilitating the clearance of misfolded protein aggregates, chronic pathological insults induce a phenotypic shift toward a dysfunctional, hyper-activated state. This sustained neurotoxic inflammation drives a vicious cycle of neuronal damage, significantly accelerating the clinical progression of the disease (11).

*Epimedium*, a widely utilized herb in traditional medicine, exhibits diverse pharmacological activities, including anti-osteoporosis, anti-tumor, antioxidant, anti-inflammatory, and immunomodulatory effects (12, 13). Beyond its well-established roles in the reproductive and cardiovascular systems, the nervous system protection of *Epimedium* and its active constituents has garnered significant attention. To comprehensively evaluate its neuroprotective profile, recent preclinical studies have highlighted its broad-spectrum efficacy across various neurodegenerative disease models (14). Specifically, it has been demonstrated to prevent dopaminergic neuron loss in Parkinson's disease (PD), alleviate autoimmune neuroinflammation in multiple sclerosis (MS), delay motor neuron degeneration in amyotrophic lateral sclerosis (ALS), and promote the clearance of mutant protein aggregates in Huntington's disease (HD) (15). Collectively, these findings underscore a multi-target therapeutic potential of *Epimedium* against severe neurodegeneration. we clarified that pharmacokinetic studies assays have confirmed that icariin (ICA)—the primary bioactive flavonoid of *Epimedium* can successfully cross the BBB (12). Previous *in vivo* and *in vitro* studies have shown that *Epimedium* and its major active constituent icariin possess neuroprotective and anti-neuroinflammatory effects (16). In APP/PS1 mice, icariin has been reported to improve cognitive function, possibly through suppression of endoplasmic reticulum stress (17). *In vitro* studies further suggest that *Epimedium* extract or icariin can alleviate microglia-mediated inflammatory responses by regulating signaling pathways related to TLR4/NF-κB, Nrf2, and the NLRP3 inflammasome (18, 19). These findings suggest that attenuation of neuroinflammation may be one of the important mechanisms by which *Epimedium* exerts its effects against AD. Among the 379 compounds identified to date, these include flavonoids, lignans, organic acids, terpenoids, hydrocarbons, dihydrophenanthrene derivatives, and alkaloids (20). While flavonoid constituents (e.g., icariin) have long been the focus of quality control and pharmacological research, non-flavonoid constituents with unique structural features in this plant have not yet received adequate attention. Notably, small-molecule terpenoids and phytosterols present in *Epimedium* have recently gained interest due to their potential neuroactive properties. Therefore, exploring these specific bioactive small molecules beyond the well-known flavonoids may open new avenues for elucidating the material basis of the neuroprotective effects of *Epimedium*.

KAT2B (also known as PCAF, P300/CBP-associated factor), a critical lysine acetyltransferase (HAT), has been demonstrated to play a central role in hippocampus-dependent memory formation and nerve growth factor-induced gene transcription (21). Existing studies indicate that AD pathological conditions are often accompanied by a global imbalance in histone acetylation levels (dysregulation of HATs/HDACs) (22). However, current research has primarily focused on isoforms such as CBP or p300. Systematic investigations regarding the changes in KAT2B expression patterns within the specific pathological microenvironment of AD, as well as the detailed mechanisms underlying its regulatory network and interactions with downstream target genes, remain lacking (23, 24).

Based on this, the present study is the first to focus on the intrinsic relationship between KAT2B and Alzheimer's disease, aiming to elucidate its molecular mechanisms in AD neurodegeneration. Although extensive research has demonstrated that *Epimedium* and its active components alleviate Alzheimer's disease through multiple mechanisms—including mitigating neuroinflammation (5), suppressing oxidative stress (25), and regulating autophagy (26),these studies have primarily centered on downstream cytoplasmic signaling events. However, existing investigations have predominantly focused on downstream cytoplasmic signaling events. The upstream epigenetic regulatory mechanisms (such as histone acetylation) that control the transcriptional initiation of these pathways remain largely unexplored. It remains unclear whether *Epimedium* may influence AD progression through specific modulation of KAT2B. Therefore, this study represents the first attempt to explore the potential role of KAT2B in the neuroprotective effects of *Epimedium*. The research aims to address a critical gap in the current literature and provide a theoretical foundation for subsequent mechanistic and translational studies.

## 2 Materials and methods

### 2.1 Predicting key targets of *Epimedium* in Alzheimer's disease

#### 2.1.1 Prediction of action targets between *Epimedium* and Alzheimer's disease (AD)

In this study, the chemical constituents of *Epimedium* were retrieved from the Traditional Chinese Medicine Systems Pharmacology Database and Analysis Platform (TCMSP). Screening was conducted based on criteria of oral bioavailability (OB) ≥ 30% and blood-brain barrier permeability (BBB) ≥ 1 to identify potential active components (27). While drug-likeness (DL) is a commonly used metric in drug screening, for natural compounds, a low DL value does not necessarily exclude their potential. Therefore, we specifically applied screening criteria focusing on OB and a significantly higher BBB permeability threshold, without incorporating DL as a filtering condition. This approach aims to avoid overlooking potential compounds with favorable absorption and high permeability (28). The molecular formulas and 3D structures of each constituent were obtained from the PubChem database and subsequently imported into the SwissTargetPrediction platform for predicting potential action targets, with the species restricted to Homo sapiens. All acquired target data were then merged, and duplicates were removed to construct the target set of *Epimedium*. AD-related targets were retrieved from the GeneCards and OMIM databases, and standardized using the UniProt database (species: Homo sapiens). The gene set obtained after integration and deduplication was defined as the AD-related target set. Furthermore, the intersection between the *Epimedium* target set and the AD target set was calculated and defined as potential key targets for *Epimedium* in the treatment of AD.

#### 2.1.2 Identification of key genes in *Epimedium* intervention for Alzheimer's disease using limma and WGCNA

AD-related transcriptome sequencing data were retrieved from the GEO database, and the original expression matrix (accession number: GSE5281) was downloaded and acquired (29). We conducted differentially expressed genes (DEGs) and weighted gene co-expression network analysis (WGCNA) on the GSE5281 dataset, which comprised superior frontal gyrus samples from Alzheimer's disease patients. DEGs between the disease and control groups were identified using the “limma” package in R (v4.4.2) software. Genes with |log2 fold change| > 1 and P.adj < 0.05 were considered significant. The “WGCNA” package was used to identify gene modules highly correlated with disease traits and to extract key genes within these modules. Outlier samples were first detected to ensure data quality. A suitable soft-thresholding power was selected to achieve a scale-free topology fit index (R^2^ ≥ 0.85), facilitating the construction of a scale-free network. The adjacency matrix was then converted into a topological overlap matrix (TOM), which quantified gene similarity based on weighted correlations with all other genes. Hierarchical clustering was performed to identify co-expression modules, with minModuleSize set to 100. Dynamic tree cutting and module merging were optimized using the mergeCutHeight parameter. Within-module connectivity was assessed using Pearson correlation coefficients. The module showing the strongest association with the disease phenotype (|correlation| highest, *P* < 0.01) was designated the key module, and its genes were extracted as core candidates for downstream functional annotation and mechanistic analysis.

#### 2.1.3 Experimental instruments

Morris Water Maze (Chengdu Taimeng Software Co., Ltd., Model: WMT-200);Ultra-Micro Full-Wavelength Microplate Reader (Thermo Fisher Scientific, USA, Model: Multiskan FC).

Multi-Sample Cryogenic Grinder (Shanghai Wanbai Biotechnology Co., Ltd., Model: Wonbio-96c); Electronic Balance (Mettler Toledo, Model: NewClassic MF MS105DU).

#### 2.1.4 Herbal materials and experimental reagents

*Epimedium* was purchased from Tongrentang Pharmacy in Guiyang City, Guizhou Province (production batch number: 24012002), and the medicinal material was authenticated by Associate Professor Yan Fulin of Guizhou University of Traditional Chinese Medicine.Hematoxylin-Eosin(HE)Stain Kit(BEIJING SOLARBBIO SCIENCE&TECHNOLOGYCO.LTD, Cat:G1120).

### 2.2 Experimental verification

#### 2.2.1 Experimental animals

Thirty-six adult male SPF-grade SD rats were purchased from the Animal Research Institute of Guizhou University of Traditional Chinese Medicine, with body weights of (180 ± 20) g. The rats were housed under normal environmental conditions: temperature (20 ± 4) °C, humidity 50%−60%, adequate ventilation, and a 12-h light/dark cycle, with free access to food and water. This animal experiment was approved by the Animal Ethics Committee of Guizhou University of Traditional Chinese Medicine (Approval No.: 20250609004). The study was conducted in accordance with the Guide for the Care and Use of Laboratory Animals published by the National Research Council of the United States (30).

#### 2.2.2 Animal grouping and drug administration

Thirty-six male Sprague-Dawley (SD) rats were intragastrically administered 200 mg/kg AlCl_3_ and intraperitoneally injected 60 mg/kg d-galactose daily for 10 weeks to establish an Alzheimer's disease (AD) model (31). Additionally, six rats were selected as a normal control group. Throughout the feeding period, a balanced indoor environment was maintained, and animals had free access to food and water. After successful modeling, the rats were randomly divided into the following groups (6 rats per group): model group, low-dose *Epimedium* group, medium-dose *Epimedium* group, high-dose *Epimedium* group, and memantine hydrochloride group. The memantine hydrochloride group received intragastric administration at a dose of 5 mg/kg. Each group was administered the aqueous extract of *Epimedium*, with high-, medium-, and low-dose groups receiving 150 mg/kg, 100 mg/kg, and 50 mg/kg, respectively. The clinical equivalent dose for the 100 mg/kg group corresponded to 685 mg/kg of the raw herbal material. The dose selection was informed by the clinical guidelines of the Chinese Pharmacopeia and relevant preclinical research on *Epimedium* and its major bioactive constituents (32). All treatments were administered once daily via intragastric gavage. After 4 consecutive weeks of administration, the rats were fasted (no food or water) for 12 h. Subsequently, anesthesia was induced by inhalation of a mixture of 2% isoflurane and oxygen at a flow rate of 0.2 ml/min. Blood was collected from the abdominal aorta, centrifuged to obtain serum, which was used for ELISA detection. Hippocampal tissues were rapidly excised on ice and stored at −80 °C for real-time quantitative PCR analysis. Brain tissues from some rats were fixed in 4% paraformaldehyde solution for pathological examination.

#### 2.2.3 Morris water maze test

The Morris water maze test was employed to investigate the spatial learning and memory abilities of SD rats in each group, which consisted of a place navigation trial and a spatial probe trial (33). A platform was placed in the first quadrant, submerged 2 cm below the water surface. In the place navigation trial, rats were randomly released into the water facing the pool wall from other quadrants. They performed this task for 5 consecutive days to locate the platform. Rats that found the platform within 90 s and remained on it for 20 s were recorded successfully. For those failing to find the platform within the time limit, they were manually guided to it and allowed to stay for 20 s. The escape latency, defined as the time taken for the rat to find the platform, was recorded. On the 6th day of the water maze test, the spatial probe trial was conducted. The platform in the first quadrant was removed. The time spent in the target quadrant within 90 s and the number of platform crossings were recorded.

#### 2.2.4 Hematoxylin and eosin (HE) staining

After the experiment, brain tissue specimens from each group were collected and fixed in 4% paraformaldehyde. The rat brain tissues were then embedded in paraffin, dewaxed, and rehydrated. Subsequently, the sections were stained with hematoxylin solution for 7 min, rinsed repeatedly with distilled water, and then stained with eosin solution for 2 min. Following dehydration and clearing, the sections were mounted with neutral resin. The morphological structure of the rat hippocampal tissue was observed and imaged under a microscope.

#### 2.2.5 ELISA

Hippocampal tissues were isolated and stored at−80 °C for subsequent analysis. According to the manufacturer's instructions of the ELISA kits, the levels of inflammatory factors IL-1β and TNF-α in the hippocampal tissues were measured.The IL-1β ELISA kit (E-EL-R0012) and TNF-α ELISA kit (E-EL-R2856) were both purchased from Wuhan Elabscience Biotechnology Co., Ltd.

#### 2.2.6 Real-time quantitative PCR (qPCR)

Frozen hippocampal tissues from rats in each group were retrieved to detect the mRNA expression levels of KAT2B, NF-κB, IL-1β, and TNF-α. Total RNA was extracted using an RNA extraction kit. After determining its concentration and purity, the RNA was reverse-transcribed into cDNA following the instructions of the reverse transcription kit. Using this cDNA as a template, real-time fluorescent quantitative PCR amplification was performed. The reaction mixture consisted of 0.4μl each of forward and reverse primers (specific sequences and product lengths are listed in the table), 1μl of cDNA template, 10μl of fluorescent dye, and 8.2μl of nuclease-free water. The reaction conditions were as follows: pre-denaturation at 95 °C for 2 min; followed by 40 cycles of denaturation at 95 °C for 15 s, annealing at 60 °C for 15 s, and extension at 72 °C for 60 s. GAPDH was used as the internal reference gene. The relative expression levels were calculated using the 2^−Δ*ΔCt*^ method.

### 2.3 Statistical analysis

Statistical analysis was performed using GraphPad Prism software (version 8.3; GraphPad Software, San Diego, CA, USA, 2020). Experimental data are presented as the mean ± standard deviation (SD). Comparisons among multiple groups were analyzed by one-way analysis of variance (ANOVA). A *P*-value of less than 0.05 (*P*<*0.05*) was considered statistically significant.

## 3 Results

### 3.1 Results of target prediction for *Epimedium* and Alzheimer's disease

**Figure 1:**
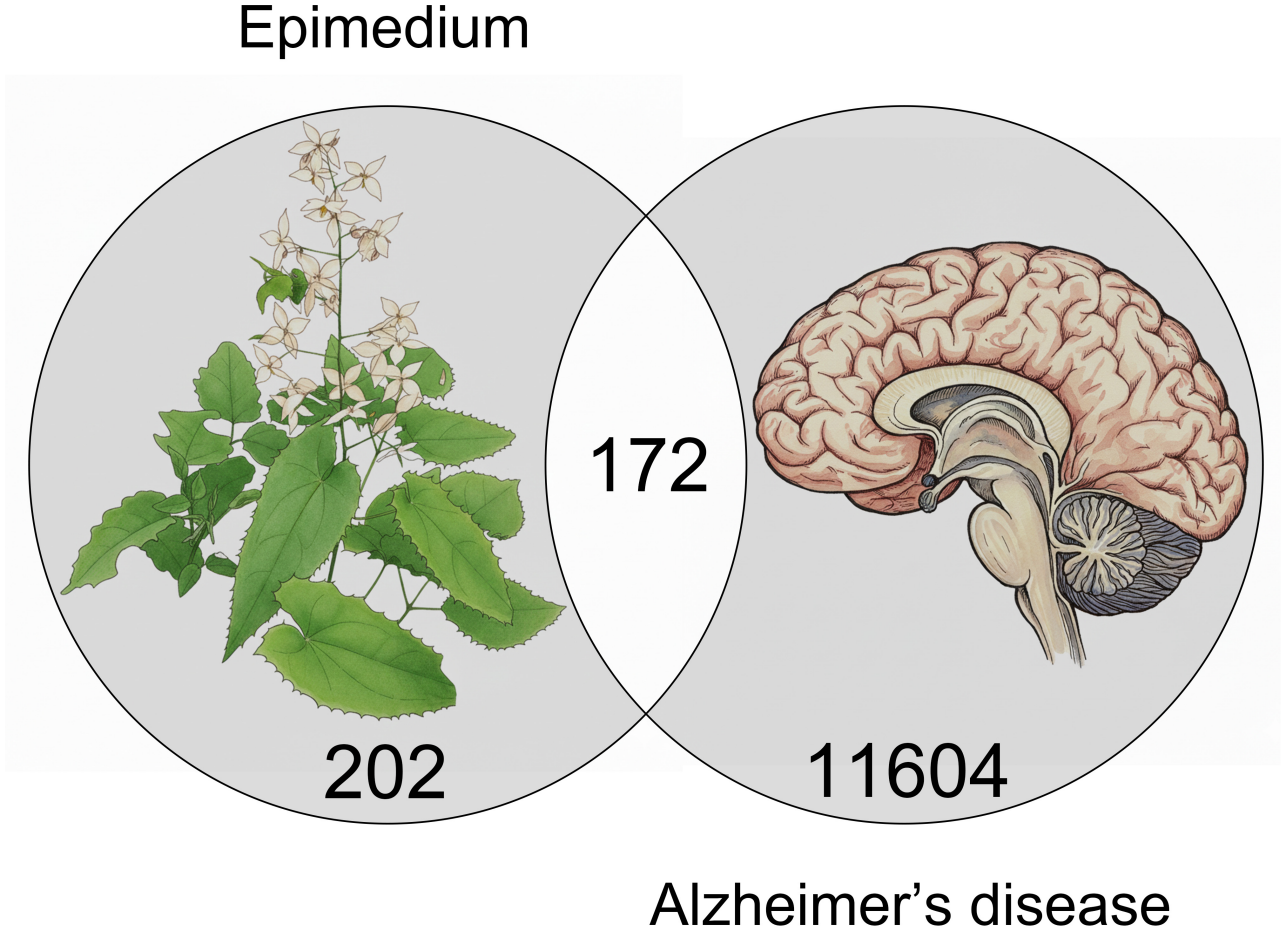
Screening of potential targets of *Epimedium* in Alzheimer's disease.

In this study, a total of 10 compounds closely associated with *Epimedium* were identified (Tables 1, 2), all of which possessed properties indicative of good absorption and blood-brain barrier permeability. Through screening, 374 potential targets of *Epimedium* (Supplementary Table S1) and 11,776 targets related to Alzheimer's disease (AD) (Supplementary Table S2) were obtained. By taking the intersection of these two target sets, 172 targets were ultimately determined as the key potential targets for *Epimedium* in the treatment of AD (Figure 1, Supplementary Table S3).

**Table 1:** Pharmacologically active compounds of Herba *Epimedium*.

NAME	CAS	Molecular Formula	OB (%)	BBB
(L)–α-terpineol	10482–56–1	C_10_H_18_O	48.79	1.72
Camphor	76–22–2	C_10_H_16_O	67.17	1.70
24–epicampesterol	4651–51–8	C_28_H_48_O	37.57	1.14
Linoleyl acetate	5999–95–1	C_20_H_36_O_2_	42.1	1.08
γ-sitosterol	83–47–6	C_29_H_50_O	36.91	1.14
Pulegone	89–82–7	C_10_H_16_O	51.59	1.73
(R)–linalool	126–91–0	C_10_H_18_O	39.8	1.36
Izosafrol	17627–76–8	C_10_H_10_O_2_	56.91	1.30
(1S,4R)–fenchone	4695–62–9	C_10_H_16_O	72.63	1.73
(-)–piperitone	4573–50–6	C_10_H_16_O	53.87	1.54

OB, oral bioavailability; BBB, blood-brain barrier; CAS, chemical abstracts service.

**Table 2:** Gene primer information.

Gene	Primer	Sequence(5^′^-3^′^)	PCR products
Rat GAPDH	Forward	GACATGCCGCCTGGAGAAAC	20bp
	Reverse	AGCCCAGGATGCCCTTTAGT	
Rat KAT2B	Forward	GTGCTACTGCAATGTACCGC	20bp
	Reverse	ATGGTGAAGACCGAGCGAAG	
Rat NF–κB	Forward	CAGACACCTTTGCACTTGGC	20bp
	Reverse	CTTGAGTAGGACCCCGAGGA	
Rat IL−1β	Forward	CCTATGTCTTGCCCGTGGAG	20bp
	Reverse	CACACACTAGCAGGTCGTCA	
Rat TNF–alpha	Forward	GGAGGGAGAACAGCAACTCC	20bp
	Reverse	GCCAGTGTATGAGAGGGACG	

### 3.2 Identification of hub genes via transcriptome analysis

**Figure 2:**
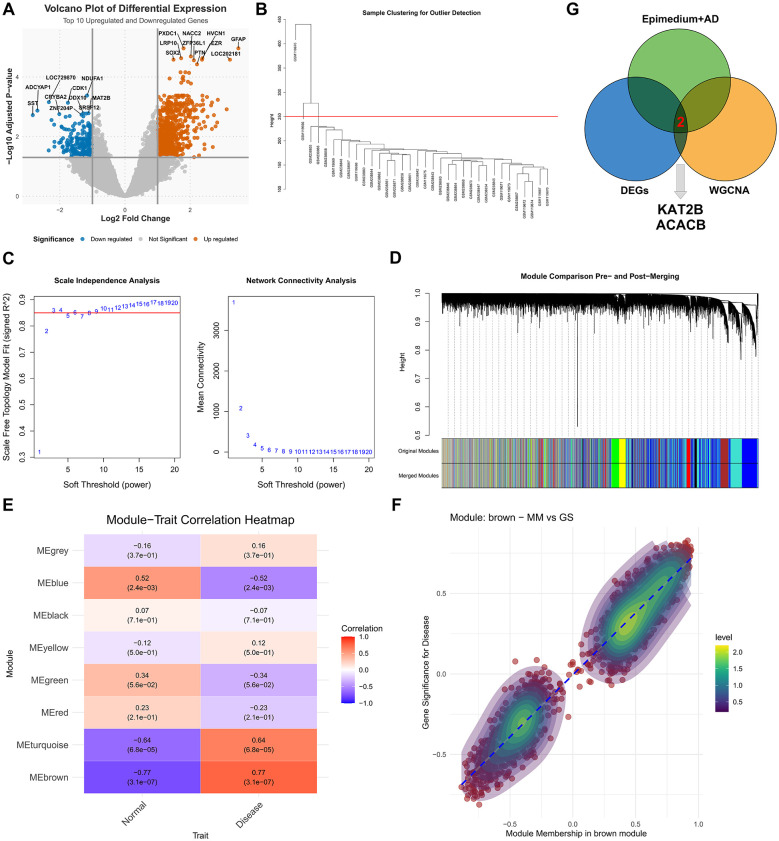
Transcriptomic analysis and Weighted Gene Co-expression Network Analysis (WGCNA) of key targets in Alzheimer‘s disease. **(A)** Volcano plot displaying the differentially expressed genes (DEGs) between the disease and control groups. The red and green dots represent significantly upregulated and downregulated genes (|log2FC| > 1, adjusted P < 0.05), respectively, with the top 10 labeled. Gray dots indicate genes with no significant difference. **(B)** Sample clustering dendrogram based on gene expression profiles to detect outliers. Colors below the dendrogram represent different experimental groups: Red (*Epimedium*+AD), Blue (DEGs analysis group), Green (identification of KAT2B/ACACB), and Purple (WGCNA analysis group). **(C)** Analysis of scale-free fit index (Signed R^2^) for various soft-threshold powers. The red line indicates the threshold (typically 0.85) for achieving a scale-free network. **(D)** Clustering dendrogram of genes based on dissimilarity measure, resulting in the assignment of co-expression modules. Each major branch in the “Height” tree represents a module. **(E)** Heatmap of the correlation between identified modules and clinical traits (Normal vs. Disease). Each cell contains the correlation coefficient and p-value. Red indicates a positive correlation with the trait, while blue indicates a negative correlation. **(F)** Scatter plot of Gene Significance (GS) for Disease vs. Module Membership (MM) in the brown module. The high correlation suggests that genes highly connected within the brown module are strongly associated with the disease phenotype.

Differential expression analysis of the GSE5281 dataset identified a total of 713 up-regulated and 248 down-regulated genes (Figure 2A, Supplementary Table S4). Subsequently, prior to performing weighted gene co-expression network analysis (WGCNA), 2 outlier samples were removed, and the remaining 32 samples (including 9 controls and 23 cases) were used to construct the co-expression network (Figure 2B). A soft threshold power of 9 was selected (scale-free topology fit index *R*^2^ = 0.85), successfully constructing a network that met the scale-free distribution criterion (Figure 2C). Based on the adjacency matrix, functional modules were identified using the dynamic tree cut method (Figure 2D), resulting in 8 distinct co-expression modules (Figure 2E). Module-trait relationship analysis revealed that the brown module exhibited the strongest correlation with the disease phenotype (Figure 2F). By evaluating the correlation between module membership (MM) and gene significance (GS), 172 hub genes were further screened from this module for subsequent in-depth analysis (Supplementary Table S5). Finally, an intersection was performed among the following gene sets: (1) the core targets derived from the intersection of compound and disease targets, (2) the up-regulated genes identified by limma analysis of the GEO dataset in the disease group, and (3) the up-regulated genes within the WGCNA module strongly associated with the disease. This intersection yielded 2 final hub genes (Figure 2G).

### 3.3 Effects on learning and memory abilities in AD rats

**Figure 3:**
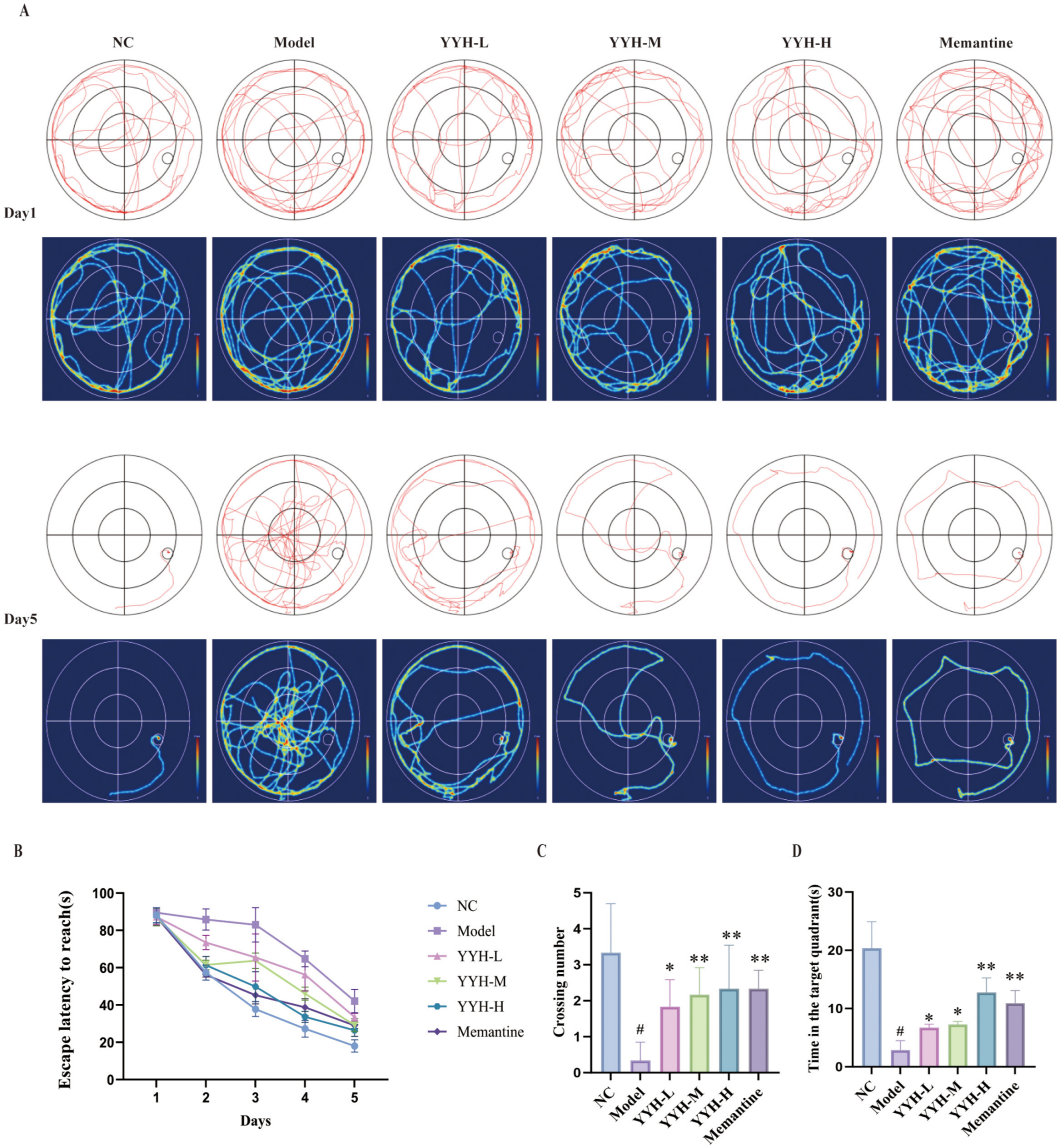
*Epimedium* ameliorates learning and memory deficits in AD rats. **(A)** Escape latency to find the hidden platform in the Morris water maze. **(B)** Escape latency to reach the platform. **(C)** Number of crossings over the exact location of the removed platform during the probe trial. Data are presented as mean ± SD. #*P* < 0.05 vs. normal control (NC) group; *P < 0.05, **P < 0.01 vs. model group. **(D)** Time spent over the exact location of the removed platform during the probe trial. Data are presented as mean ± SD. #*P* < 0.05 vs. NC group; *P < 0.05, **P < 0.01 vs. model group.

**Figure 4:**
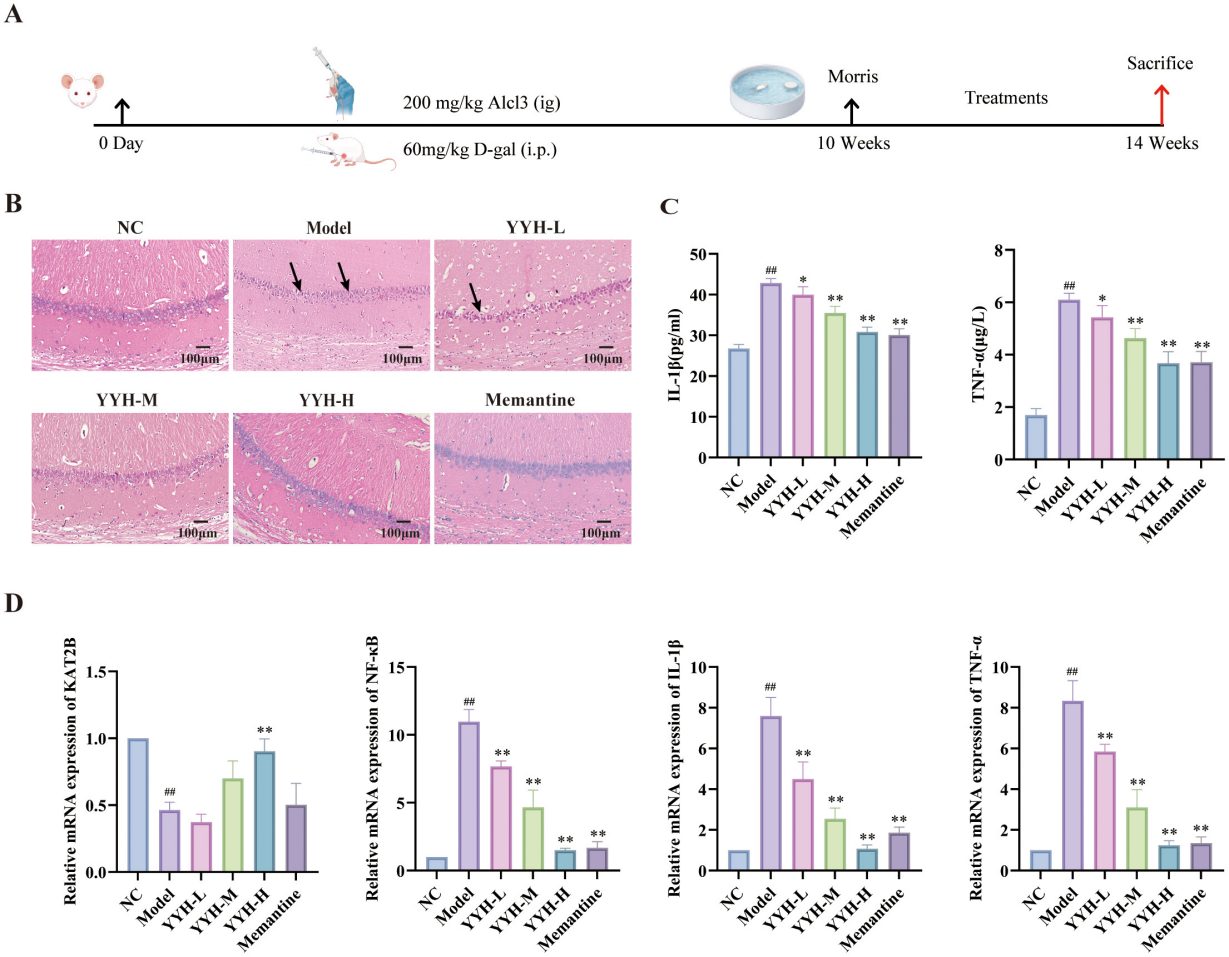
Effects of *Epimedium*(YYH) on brain histopathology and inflammatory factor profiles in AlCl3/D-gal-induced Alzheimer's disease (AD) model rats. **(A)** Schematic representation of the experimental timeline, model establishment, and drug administration protocol. Mice were intraperitoneally injected with D-galactose (D-gal, 60 mg/kg) and intragastrically administered aluminum chloride (AlCl3, 200 mg/kg) daily from day 0 for 14 consecutive weeks to establish an AD-like model. From week 10 onwards, the YYH low-dose (YYH-L), medium-dose (YYH-M), and high-dose (YYH-H) groups, as well as the positive control Memantine group, received corresponding drug interventions until the termination of the experiment at week 14. **(B)** Representative photomicrographs of hematoxylin-eosin (HE) staining showing pathomorphological alterations in the hippocampal region of mouse brain tissue across all groups (scale bar = 100 μm). **(C)** Serum levels of pro-inflammatory cytokines interleukin-1β (IL-1β) and tumor necrosis factor-α (TNF-α) in mice from each group. Data are presented as mean ± SD. ##*P* < 0.01 vs. normal control (NC) group; *P < 0.05, **P < 0.01 vs. model group. **(D)** Relative mRNA expression levels of kynurenine aminotransferase 2B (KAT2B), nuclear factor kappa B (NF-κB), IL-1β, and TNF-α in mouse brain tissue. Data are presented as mean ± SD. ##*P* < 0.01 vs. NC group; **P < 0.01 vs. model group.

Compared with the control group, rats in the AD model group exhibited a significant increase in escape latency (*P* < 0.05) (Figures 3A, B), a significant reduction in the number of platform crossings (*P*<*0.05*) (Figure 3C), and a significant decrease in the time spent in the target quadrant (*P*<*0.05*) (Figure 3D). However, all dose groups of *Epimedium* reversed these deficits to varying degrees, with the high-dose group showing the most pronounced improvement. Specifically, compared with the model group, rats in all *Epimedium* treated groups showed significantly shortened escape latency (*P* < 0.05), significantly increased time spent in the target quadrant (*P* < 0.05), and significantly increased numbers of platform crossings (*P* < 0.05), with the high-dose group demonstrating the greatest improvement. The overall workflow of the animal experiment is shown in Figure 4A.

### 3.4 Histopathological analysis of rat hippocampus by HE staining

Histopathological examination of rat hippocampal tissue under light microscopy revealed the following: In the control group, neuronal cells exhibited intact morphology and were arranged in an orderly manner. In contrast, the AD model group displayed a disorganized hippocampal structure characterized by irregularly sized and arranged neurons, along with the presence of pyknotic nuclei and a noticeable reduction in neuronal number. Notably, treatment with *Epimedium* led to improved neuronal damage and cellular morphology. Neurons in the *Epimedium*-treated groups showed more preserved structure and relatively uniform arrangement. Specifically, a significant improvement in the neuronal damage was observed in the hippocampal region of rats receiving the high-dose *Epimedium* intervention (Figure 4B).

Based on the findings above, *Epimedium* intervention demonstrated a neuroprotective effect in the AD rat model. This effect was specifically manifested as an effective alleviation of the degree of neuronal damage and a significant promotion of neuronal survival.

### 3.5 Enzyme-linked immunosorbent assay (ELISA)

Compared with the blank control group, the levels of IL-1β and TNF-α in the model group were significantly increased (*P* < 0.05). In comparison with the model group, all *Epimedium*-treated groups exhibited significant reductions in the levels of IL-1β and TNF-α, with the most pronounced decrease observed in the high-dose *Epimedium* group (*P* < 0.05) (Figure 4C).

### 3.6 Quantitative real-time PCR (qRT-PCR)

Compared with the blank control group, the model group exhibited significantly decreased mRNA levels of KAT2B and significantly increased mRNA levels of NF-κB, IL-1β, and TNF-α (*P* < 0.05). In contrast, all *Epimedium* treatment groups showed markedly elevated mRNA levels of KAT2B and reduced mRNA levels of NF-κB, IL-1β, and TNF-α compared to the model group (*P* < 0.05). Notably, the most pronounced effects were observed in the high-dose *Epimedium* group, which demonstrated the greatest increase in KAT2B mRNA and the most significant decrease in NF-κB, IL-1β, and TNF-α mRNA levels (*P* < 0.05) (Figure 4D).

## 4 Discussion

Currently, clinical treatment strategies for Alzheimer's disease (AD) still face significant limitations, necessitating the urgent development of novel therapeutic interventions. Traditional Chinese Medicine (TCM), with its multi-component and multi-target characteristics, demonstrates unique advantages in treating neurodegenerative diseases. However, its precise molecular mechanisms remain elusive. This study integrates modern network pharmacology and bioinformatics approaches to systematically decode the underlying mechanisms of *Epimedium* (a TCM herb) in intervening AD. During preliminary screening, our focus turned to Acetyl-CoA Carboxylase Beta (ACACB). Although ACACB, as the rate-limiting enzyme for mitochondrial fatty acid oxidation, plays a crucial role in regulating energy homeostasis (34) and can effectively inhibit fatty acid oxidation (35–37), it is more often regarded as a downstream marker of metabolic dysregulation (38). This makes it difficult to directly explain the upstream core pathologies in AD, such as abnormal protein aggregation, synaptic loss, and neuroinflammation. Consequently, this study strategically shifts its focus to the histone acetyltransferase KAT2B (PCAF), positioned upstream in the regulatory network, aiming to reveal the neuroprotective mechanism of *Epimedium* from an epigenetic perspective.

This study validated the therapeutic potential of *Epimedium* for AD model rats through *in vivo* experiments. The D-galactose/AlCl_3_ induced rat model used in this study is a commonly used chemical model that reproduces several AD changes, including cognitive deficits, oxidative stress-related injury, and neuropathological abnormalities. Chronic D-galactose exposure is mainly linked to aging-related oxidative stress (39), while aluminum exposure contributes to neurotoxic and inflammatory changes (40). These features provide a relevant experimental context for evaluating the neuroprotective effects of *Epimedium* in AD pathology. The Morris water maze test results showed that *Epimedium* intervention significantly shortened the escape latency of AD rats and improved their spatial learning and memory abilities. Histopathological examination further confirmed that in the model group, a large number of hippocampal neurons were lost, with disordered arrangement and pyknotic cell bodies. In contrast, the *Epimedium* treatment group (especially the high-dose group) showed significant improvement in neuronal damage, with intact cellular morphology and structure, and a more regular cellular arrangement. In this study, the hippocampus was selected for pathological evaluation because it is highly vulnerable in AD and closely related to the spatial cognitive function assessed by the Morris water maze. Although other brain regions were not examined, the protective effects of *Epimedium* on hippocampal neurons and cognitive performance suggest its potential neuroprotective role.

To elucidate the molecular basis of the aforementioned therapeutic effects, we further investigated the interaction between KAT2B and the neuroinflammation axis. Previous studies have shown that KAT2B is a lysine acetyltransferase highly expressed in the brain, playing a crucial role in maintaining normal neural function, chromatin remodeling, and transcriptional regulation (41, 42). Particularly noteworthy is the research by Maurice et al. (22), which confirmed that mice with knockout of the KAT2B gene exhibited significant short-term memory deficits and alterations in hippocampal microstructure, displaying age-dependent memory decline. This directly reveals the core protective role of KAT2B in maintaining cognitive function.

In line with this theory, qPCR detection in this study found that the mRNA expression level of KAT2B in the hippocampal tissue of AD model rats was significantly reduced, which may be a key epigenetic factor leading to their cognitive impairment (43). Simultaneously, the mRNA and protein levels (ELISA) of the transcription factor NF-κB and its downstream inflammatory factors (IL-1β, TNF-α) were abnormally elevated in the model group. This pathological phenomenon aligns with the typical characteristics of chronic neuroinflammation in AD (44), namely the sustained activation of NF-κB in cortical and hippocampal glutamatergic neurons (45, 46). After intervention with *Epimedium*, the expression level of KAT2B in the rat hippocampus was significantly restored (increased), while the levels of NF-κB, IL-1β, and TNF-α were synchronously reduced. This inverse correlation suggests that *Epimedium* may restore the normal expression of KAT2B, reshape the histone acetylation environment in the hippocampal region, thereby enhancing neural plasticity. Concurrently, the treatment effectively blocked the NF-κB-driven inflammatory cascade. Although researchers (47) have pointed out that certain HAT family members (such as p300) can form complexes with NF-κB to promote inflammation, the data from this study support the view that KAT2B primarily exerts a neuroprotective function in the context of AD. The deficiency of KAT2B may increase neuronal susceptibility to inflammatory damage (48), while *Epimedium* restores the balance of “neuroprotection-immune homeostasis” by upregulating KAT2B and inhibiting the NF-κB pathway.

In summary, this study not only confirmed the therapeutic efficacy of *Epimedium* in ameliorating cognitive deficits and pathological damage in AD, but also innovatively revealed its underlying molecular mechanism. Specifically, *Epimedium* likely exerts its effects by upregulating the expression of the core epigenetic enzyme KAT2B while simultaneously inhibiting the NF-κB-mediated neuroinflammatory response. This finding provides a novel epigenetic perspective for understanding the “multi-component, multi-target” mechanism of *Epimedium* against AD.

## 5 Conclusion

This study preliminarily explored the therapeutic role of *Epimedium* in Alzheimer's disease and identified KAT2B as a potential core target for modulating AD. By mediating NF-κB and its related factors, KAT2B likely contributes to the underlying mechanism through which *Epimedium* improves Alzheimer's pathology, providing theoretical guidance for further in-depth research in this field.

## Data Availability

The raw data supporting the conclusions of this article will be made available by the authors, without undue reservation.
